# Thyroid cancer trends in China and its comparative analysis with G20 countries: Projections for 2020–2040

**DOI:** 10.7189/jogh.14.04131

**Published:** 2024-06-14

**Authors:** Yi Gong, Qin Jiang, Mimi Zhai, Tenglong Tang, Sushun Liu

**Affiliations:** 1 Department of General Surgery, the Second Xiangya Hospital, Central South University, Changsha, Hunan, China; 2 Clinical Nursing Teaching and Research Section, the Second Xiangya Hospital, Central South University, Changsha, Hunan, China; 3 School of Nursing, Hunan University of Chinese Medicine, Changsha, Hunan, China

## Abstract

**Background:**

Thyroid cancer, a leading type of endocrine cancer, accounts for 3–4% of all cancer diagnoses. This study aims to analyse and compare thyroid cancer patterns in China and the Group twenty (G20) countries, and predict these trend for the upcoming two decades.

**Methods:**

This observational longitudinal study utilised data from the Global Burden of Disease (GBD) study 2019. We used metrics including incidence, mortality, mortality-incidence ratio (MIR), age-standardised rate (ASR) and average annual percent change (AAPC) to examine thyroid cancer trends. Joinpoint regression analysis was used to identify periods manifesting notable changes. The association between sociodemographic index (SDI) and AAPC were investigated. The autoregressive integrated moving average (ARIMA) model was used to predict thyroid cancer trends from 2020 to 2040.

**Results:**

From 1990 to 2019, thyroid cancer incidence cases in China increased by 289.6%, with a higher AAPC of age-standardised incidence rate (ASIR) in men. Contrastingly, the G20 demonstrated a smaller increase, particularly among women over 50. Despite the overall age-standardised mortality rate (ASMR) was higher in the G20, the increase in mortality was less pronounced than in China. Age-standardised incidence rate increased across all age groups and genders, with a notable rise among men aged 15–49. ASMR decreased in specific age groups and genders, especially among women. Conversely, the ASMR significantly increased in group aged over 70. The MIR exhibited a declining trend, but this decrease was less noticeable in men and the group aged over 70. Joinpoint analysis pinpointed significant shifts in overall ASIR and ASMR, with the most pronounced increase in ASIR during 2003–2011 in China and 2003–2010 in the G20. Predictions suggested a continual ASIR uptrend, especially in the 50–69 age group, coupled with a predicted ASMR downturn among the elderly by 2040. Moreover, the proportion of thyroid cancer deaths attributable to high body mass index (BMI) escalated, with significant increase in Saudi Arabia and a rise to 7.4% in China in 2019.

**Conclusions:**

Thyroid cancer cases in incidence and mortality are escalating in both China and the G20. The increasing trend may be attributed to factors beyond overdiagnosis, including environmental and genetic factors. These findings emphasise the necessity for augmenting prevention, control, and treatment strategies. They also highlight the significance of international collaboration in addressing the global challenge posed by thyroid cancer.

Thyroid cancer is a well-recognised subtype of endocrine cancer, prominently positioned among head and neck malignancies, and accounting for approximately three to four percent of all cancer cases [1,2]. Although the median age for thyroid cancer diagnosis is in the early 50s, it is the most prevalent cancer among adolescents and adults aged 16–33 [3,4]. The incidence of thyroid cancer has increased by 313% during the past 40 years [3,5]. While its incidence demonstrates variability worldwide, a discernible upward trend has been observed in numerous countries, including the United States, China, and Canada [6]. Although various studies have provided data on thyroid cancer trend in China, there remains a noticeable gap in comparative study between China and other countries. Such a comparative analysis is crucial for China to identify areas for improvement and to adopt successful practices from other countries [7,8].

The Global Burden of Disease (GBD) study 2019, a publicly accessible database, offers as a comprehensive repository of data on the burden of 354 diseases and injuries worldwide. Analysing the GBD study allows for the identification of emerging trends and shifts in thyroid cancer. Previous studies have primarily focused on the global trends of thyroid cancer [7,9], as well as specific analyses pertaining to China [8,10,11]. Additionally, comparative studies have been conducted to analyse thyroid cancer trend across various sociodemographic index (SDI) regions, contrasting China with the United States, within the European Union, and among a selection of ten countries [12–15]. Presently, there is a limited number of studies that compare the trends of thyroid cancer in China with those in other countries. This scarcity of comparative research impedes China's ability to pinpoint deficiencies in its own health policies and practices, and limits learning from the effective strategies and experiences of other countries.

The Group of Twenty (G20) is an international economic cooperation forum that includes a mix of developed and developing countries [16]. It provides valuable research insights relevant to China, as China's average population age is lower than that of most G20 countries [17]. Comparisons between China and G20 countries offer insights into China’s standing relative to this set of developed or rapidly developing countries [18–21]. Consequently, the study is designed to conduct a comprehensive comparative and predictive analysis of thyroid cancer between China and the G20 countries, utilising the GBD study and focusing on factors such as incidence, mortality, and MIR across different age groups, genders and regions. The study is poised to reveal significant insights into China's successes and areas needing improvement in epidemic control. Furthermore, it presents an opportunity to learn from the successful strategies implemented by other G20 countries.

## METHODS

### Data source

The foundational data for our study was derived from the GBD Study 2019. In the GBD study, thyroid cancer was defined as a malignant tumour originating from either thyroid follicular epithelial cells or parafollicular cells. It was further categorised into various types, including papillary carcinoma, follicular carcinoma, poorly differentiated carcinoma, undifferentiated carcinoma, and medullary carcinoma. The diagnosis codes of thyroid cancer used in our study included C73-C73.9, D09.3, D09.8, D34-D34.9, D44.0, Z85.850 in ICD-10 and 193-193.9, 226-226.9 in ICD-9. It was pivotal to note that our study meticulously ensured that no data compromising patient privacy or revealing personally identifiable information was involved. All pertinent legal and ethical guidelines were adhered to in utilising GBD data, in compliance with the Creative Commons Attribution-NonCommercial-NoDerivatives 4.0 International License and Section 7 of the University of Washington's Website Terms and Conditions of Use.

### Mortality-to-incidence ratio

The mortality-to-incidence ratio (MIR) provides insightful perspectives into disease trend and acted as a metric for evaluating health care systems. It is derived from the age-standardised mortality rate (ASMR) and age-standardised incidence rate (ASIR) obtained through the GBD study [22]. MIR provides a straightforward calculation method using existing cancer registry data. It is particularly effective in assessing the efficiency of health care in cancer treatment and management [7,14].

### Sociodemographic index

The sociodemographic index (SDI) is an index that evaluates regional development, taking into account factors including fertility rate, lagged distributed income per capita, and mean years of education for individuals aged over 15. The SDI data, which ranged from 0 to 1, is sourced from the Institute for Health Metrics and Evaluation.

### Trend prediction

The ASIR and ASMR were predicted utilising an ARIMA model from 2020 to 2040 [23,24]. Population data utilised in our analysis for the same timeframe was obtained from the United Nations Department of Economic and Social Affairs Population Division. A comprehensive description of the methodology could be found in our prior study [25]. The detailed method was as follows: the ARIMA model, a method for forecasting time series data, combines autoregressive (AR) and moving average (MA) elements with differencing (d) to stabilise the series. In the ARIMA (p, d, q) model, 'p' indicates the autoregressive terms count, 'd' denotes differencing order, and 'q' signifies moving average terms. This research employed xtarima function of STATA to select the best ARIMA(p,d,q) for projecting disease incidence and mortality trends from 2020 to 2040.

### Statistical analysis

The cases of incidence, mortality, disability-adjusted life years (DALY) and MIR were used to assess the severity of thyroid cancer at specific time points. In addition, ASIR, ASMR, ASDR and ASR of MIR were employed to describe the trend from 1990 to 2019. Joinpoint regression analysis was applied to examine the long-term trend with significant changes in thyroid cancer in China and G20. The joinpoint regression analysis began by fitting the simplest model with no joinpoints and systematically tested for the need to add more joinpoints through statistical methods. In this study, the number of joinpoints was optimised using a grid search approach, and their significance was verified using the Monte Carlo permutation method [26]. The AAPC calculated from the final model determines the trend direction: an AAPC with a lower 95% confidence interval (CI) above zero signals an increasing trend, whereas an AAPC with an upper 95% CI below zero indicates a decreasing trend. A 95% CI that encompasses zero suggests a stable trend. The correlation between AAPC and SDI was examined via Pearson correlation analysis. Data analysis in our study was conducted using Stata 17.0, and a *P*-value of <0.05 was considered to be statistically significant.

## RESULTS

### Overall trend of thyroid cancer in China and the G20 countries

**Figure 1:**
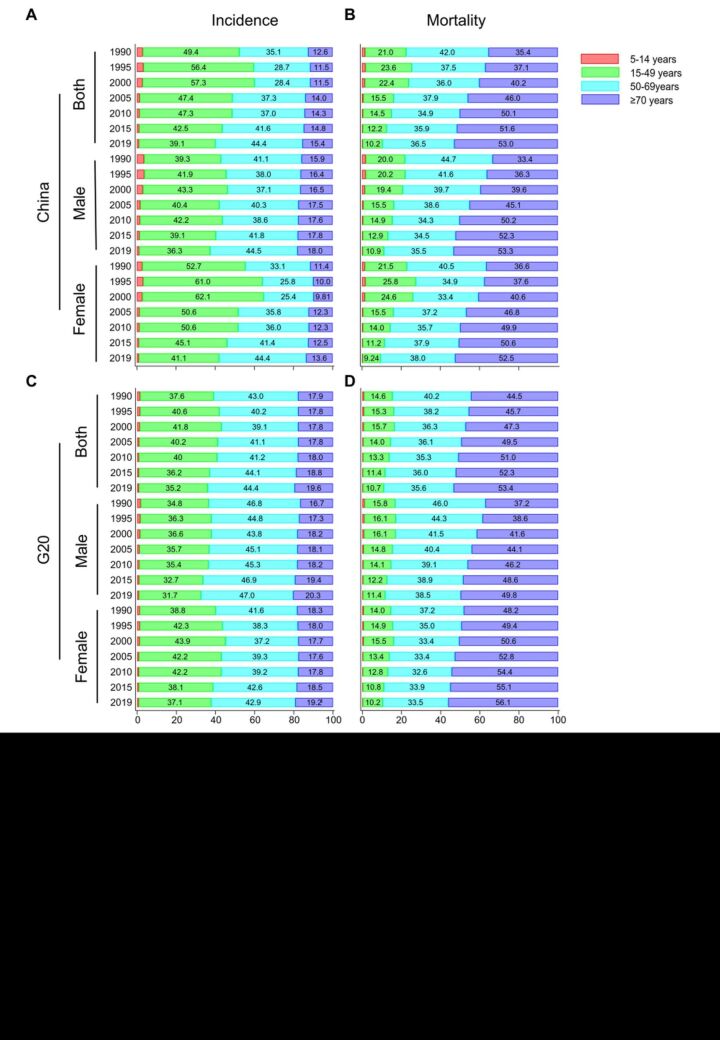
The age distribution for incidence and mortality of thyroid cancer. **Panel A.** Age distribution of incidence in China for genders. **Panel B.** Age distribution of mortality in China for genders. **Panel C.** Age distribution of incidence in the G20 countries for genders. **Panel D.** Age distribution of mortality in the G20 countries for genders. G20 **–** Group of Twenty

The incidence case in China witnessed a substantial increase from 10 030 cases in 1990 to 39 080 cases in 2019, marking a remarkable rise of 289.6% (**Table 1**, **Figure 1**, panel A). The AAPC of ASIR reached a noteworthy 4.10 (95% uncertainty interval (UI) = 3.80, 4.50) in China, with a more pronounced increase observed in men, where the AAPC reached 5.90 (95% UI = 5.40, 6.40). In comparison, although the G20 countries also demonstrated an increase in incidence cases, the AAPC was lower than that in China, measuring 2.20 (95% UI = 2.10, 2.30). Notably, the AAPC of ASIR in women within the G20 countries was significantly lower, with a value of 1.80 (95% UI = 1.70, 1.90), especially in the group aged over 50 (**Table 1**, **Figure 1**, panel C).

**Table 1:** The incidence cases, age-standardised rates, and temporal trend of thyroid cancer from 1990 to 2019

Characteristics	1990		2019		1990–2019
	**Incidence cases**	**ASIR per 100 000**	**Incidence cases**	**ASIR per 100 000**	**AAPC**
	**No. ×10^3^ (95% UI)**	**No. (95% UI)**	**No. ×10^3^ (95% UI)**	**No. (95% UI)**	**No. (95% UI)**
China*					
Both	10.03 (8.40, 11.91)	0.85 (0.71, 1.01)	39.08 (32.28, 47.66)	2.75 (2.27, 3.35)	4.10 (3.80, 4.50)
*5–14*	0.30 (0.21, 0.35)	0.14 (0.10, 0.17)	0.40 (0.33, 0.46)	0.28 (0.23, 0.32)	2.40 (1.60, 3.20)
*15–49*	4.96 (3.85, 6.01)	0.74 (0.58, 0.90)	15.28 (12.45, 19.02)	2.12 (1.73, 2.64)	3.80 (3.00, 4.60)
*50–69*	3.52 (2.96, 4.29)	2.28 (1.92, 2.78)	17.37 (14.28, 21.20)	4.71 (3.87, 5.75)	2.40 (2.10, 2.80)
*≥70*	1.26 (1.09, 1.73)	3.29 (2.84, 4.52)	6.02 (4.98, 7.00)	5.58 (4.61, 6.48)	1.80 (1.50, 2.10)
Male	2.51 (2.04, 3.10)	0.41 (0.33, 0.51)	16.11 (12.08, 20.19)	2.22 (1.67, 2.79)	5.90 (5.40, 6.40)
*5–14*	0.09 (0.07, 0.11)	0.09(0.07, 0.10)	0.19 (0.13, 0.23)	0.24 (0.17, 0.30)	3.60 (3.00, 4.20)
*15–49*	0.99 (0.80, 1.20)	0.29 (0.23, 0.35)	5.85 (4.51, 7.35)	1.58 (1.22, 1.99)	6.00 (5.40, 6.60)
*50–69*	1.03 (0.82, 1.30)	1.29 (1.03, 1.63)	7.18 (5.32, 9.24)	3.89 (2.88, 5.01)	3.90 (3.60, 4.10)
*≥70*	0.40 (0.33, 0.51)	2.42 (2.01, 3.10)	2.90 (2.17, 3.56)	5.89 (4.41, 7.23)	3.10 (2.50, 3.70)
Female	7.53 (5.91, 9.31)	1.31 (1.03, 1.62)	22.97 (17.46, 30.16)	3.29 (2.50, 4.32)	3.20 (2.90, 3.60)
*5–14*	0.21 (0.12, 0.25)	0.21 (0.12, 0.25)	0.22 (0.19, 0.25)	0.33 (0.29, 0.39)	1.70 (0.40, 3.10)
*15–49*	3.97 (2.87, 5.01)	1.23 (0.89, 1.55)	9.44 (6.96, 12.87)	2.69 (1.98, 3.67)	2.60 (1.60, 3.60)
*50–69*	2.49 (1.98, 3.16)	3.35 (2.66, 4.26)	10.19 (7.80, 13.30)	5.53 (4.23, 7.21)	1.70 (1.20, 2.20)
*≥70*	0.86 (0.71, 1.23)	3.96 (3.27, 5.65)	3.12 (2.46, 3.94)	5.31 (4.18, 6.71)	1.00 (0.70, 1.30)
**G20**					
Both	68.11 (64.71, 71.89)	1.84 (1.75, 1.94)	168.51 (155.10, 181.36)	3.46 (3.19, 3.73)	2.20 (2.10, 2.30)
*5–14*	0.99 (0.86, 1.08)	0.14 (0.12, 0.15)	1.29 (1.19, 1.42)	0.19 (0.18, 0.21)	1.20 (0.80, 1.50)
*15–49*	25.64 (23.54, 27.68)	1.32 (1.21, 1.43)	59.39 (53.82, 65.19)	2.40 (2.17, 2.63)	2.10 (1.80, 2.30)
*50–69*	29.31 (28.10, 30.71)	5.64 (5.41, 5.91)	74.77 (68.90, 80.46)	7.31 (6.74, 7.87)	0.90 (0.70, 1.00)
*≥70*	12.16 (11.33, 13.02)	7.69 (7.16, 8.23)	33.06 (29.08, 35.78)	9.00 (7.92, 9.74)	0.50 (0.40, 0.60)
Male	19.18 (18.09, 20.36)	1.03 (0.97, 1.09)	58.61 (52.36, 64.52)	2.39 (2.14, 2.64)	3.00 (2.80, 3.10)
*5–14*	0.34 (0.30, 0.37)	0.09 (0.08, 0.10)	0.51 (0.45, 0.58)	0.15 (0.13, 0.16)	1.60 (1.20, 2.00)
*15–49*	6.67 (6.23, 7.15)	0.67 (0.63, 0.72)	18.60 (16.55, 20.54)	1.47 (1.31, 1.62)	2.70 (2.60, 2.90)
*50–69*	8.97 (8.34, 9.54)	3.50 (3.25, 3.72)	27.57 (24.37, 30.78)	5.49 (4.85, 6.13)	1.50 (1.40, 1.70)
*≥70*	3.20 (3.00, 3.46)	5.07 (4.77, 5.49)	11.92 (10.33, 13.08)	7.44 (6.44, 8.16)	1.30 (1.10, 1.50)
Female	48.93 (45.59, 52.77)	2.67 (2.49, 2.88)	109.91 (99.16, 120.57)	4.55 (4.10, 4.99)	1.80 (1.70, 1.90)
*5–14*	0.65 (0.54, 0.73)	0.19 (0.16, 0.21)	0.78 (0.71, 0.86)	0.24 (0.22, 0.27)	0.90 (0.50, 1.30)
*15–49*	18.97 (16.95, 20.96)	2.00 (1.79, 2.21)	40.79 (35.74, 45.94)	3.36 (2.94, 3.78)	1.80 (1.50, 2.10)
*50–69*	20.34 (19.30, 21.56)	7.72 (7.33, 8.19)	47.20 (42.94, 52.01)	9.08 (8.26, 10.01)	0.50 (0.40, 0.70)
*≥70*	8.97 (8.28, 9.71)	9.43 (8.70, 10.21)	21.14 (18.22, 23.24)	10.21 (8.80, 11.22)	0.30 (0.20, 0.40)

*Expressed in years.

AAPC – average annual percent change, ASIR – age-standardised incidence rate, G20 – Group of Twenty, UI – uncertainty interval

The mortality case of thyroid cancer in China was 7240 cases in 2019, representing an increase of 118.1%, while the G20 had a 47.3% increase with 31 450 cases (**Table 2**, **Figure 1**, panels B, D). Interestingly, the G20 countries demonstrated a lower increase in mortality cases, despite having a higher overall ASMR than China for both 1990 and 2019. Although mortality cases increased in both China and the G20 countries, declining trends in ASMR were observed across specific age groups and genders (**Table 2**, **Figure 1**, panels B, D). Additionally, the AAPC of ASMR was lower in women both in China and the G20 countries (**Table 2**). Similar to the AAPC of ASMR, the overall trend of ASDR increased during the period, but it decreased in women within certain age groups, and even in a few age groups among men, both in China and the G20 countries (**Table 3**). The MIR was 0.19 in China and the G20 in 2019. The AAPC was −2.00 (95% UI = −2.20, −1.80) in China, while for the G20 countries, it was −0.9 (95% UI = −1.00, −0.90) (**Table 4**).

**Table 2:** The mortality cases, age-standardised rates, and temporal trend of thyroid cancer from 1990 to 2019

Characteristics	1990		2019		1990–2019
	**Mortality cases**	**ASMR per 100 000**	**Mortality cases**	**ASMR per 100 000**	**AAPC**
	**No. ×10^3^ (95% UI)**	**No. (95% UI)**	**No. ×10^3^ (95% UI)**	**No. (95% UI)**	**No. (95% UI)**
China*					
Both	3.32 (2.86, 4.13)	0.28 (0.24, 0.35)	7.24 (6.01, 8.48)	0.51 (0.42, 0.60)	2.10 (2.00, 2.20)
*5–14*	0.05 (0.04, 0.06)	0.03 (0.02, 0.03)	0.02 (0.02, 0.02)	0.02 (0.01, 0.02)	−1.70 (−2.70, −0.60)
*15–49*	0.70 (0.56, 0.84)	0.10 (0.08, 0.13)	0.74 (0.60, 0.88)	0.10 (0.08, 0.12)	0.00 (-0.80, 0.80)
*50–69*	1.40 (1.18, 1.75)	0.91 (0.76, 1.13)	2.65 (2.18, 3.18)	0.72 (0.59, 0.86)	−0.80 (−1.10, −0.60)
*≥70*	1.18 (1.02, 1.59)	3.07 (2.68, 4.15)	3.84 (3.20, 4.44)	3.55 (2.96, 4.11)	0.50 (0.40, 0.60)
Male	1.23 (1.00, 1.52)	0.20 (0.16, 0.25)	4.21 (3.18, 5.24)	0.58 (0.44, 0.72)	3.70 (3.20, 4.10)
*5–14*	0.02 (0.02, 0.03)	0.02 (0.02, 0.03)	0.01 (0.01, 0.02)	0.02 (0.01, 0.02)	−0.70 (−1.00, −0.30)
*15–49*	0.25 (0.20, 0.30)	0.07 (0.06, 0.09)	0.46 (0.34, 0.58)	0.12 (0.09, 0.16)	1.90 (1.50, 2.30)
*50–69*	0.55 (0.44, 0.69)	0.69 (0.55, 0.87)	1.50 (1.12, 1.92)	0.81 (0.60, 1.04)	0.60 (0.20, 0.90)
*≥70*	0.41 (0.34, 0.54)	2.49 (2.08, 3.30)	2.25 (1.71, 2.73)	4.56 (3.47, 5.54)	2.20 (1.70, 2.70)
Female	2.09 (1.71, 2.68)	0.36 (0.30, 0.47)	3.03 (2.40, 3.76)	0.43 (0.34, 0.54)	0.60 (0.50, 0.70)
*5–14*	0.03 (0.02, 0.04)	0.03 (0.02, 0.04)	0.01 (0.01, 0.01)	0.01 (0.01, 0.01)	−2.90 (−4.30, −1.60)
*15–49*	0.45 (0.33, 0.58)	0.14 (0.10, 0.18)	0.28 (0.21, 0.38)	0.08 (0.06, 0.11)	−2.00 (−2.90, −1.20)
*50–69*	0.85 (0.67, 1.11)	1.14 (0.91, 1.50)	1.15 (0.90, 1.46)	0.62 (0.49, 0.79)	−2.10 (−2.50, −1.70)
*≥70*	0.76 (0.64, 1.08)	3.51 (2.94, 4.98)	1.59 (1.26, 1.96)	2.71 (2.15, 3.34)	−0.90 (−1.10, −0.80)
**G20**					
Both	16.59 (15.61, 18.26)	0.45 (0.42, 0.49)	31.45 (28.40, 33.88)	0.65 (0.58, 0.70)	1.30 (1.20, 1.30)
*5–14*	0.13 (0.11, 0.14)	0.02 (0.01, 0.02)	0.08 (0.08, 0.10)	0.01 (0.01, 0.01)	−1.20 (−1.50, −0.80)
*15–49*	2.42 (2.13, 2.77)	0.12 (0.11, 0.14)	3.36 (3.01, 3.71)	0.14 (0.12, 0.15)	0.30 (0.10, 0.50)
*50–69*	6.66 (6.29, 7.27)	1.28 (1.21, 1.40)	11.21 (10.26, 12.08)	1.10 (1.00, 1.18)	−0.50 (−0.70, −0.40)
*≥70*	7.38 (6.80, 8.15)	4.67 (4.30, 5.15)	16.80 (14.55, 18.06)	4.57 (3.96, 4.92)	−0.10 (−0.10, 0.00)
Male	5.58 (5.18, 6.06)	0.30 (0.28, 0.32)	13.54 (12.03, 14.88)	0.55 (0.49, 0.61)	2.10 (2.10, 2.20)
*5–14*	0.06 (0.05, 0.07)	0.02 (0.01, 0.02)	0.05 (0.04, 0.05)	0.01 (0.01, 0.01)	−0.80 (−1.20, −0.40)
*15–49*	0.88 (0.80, 0.99)	0.09 (0.08, 0.10)	1.54 (1.35, 1.72)	0.12 (0.11, 0.14)	1.10 (0.90, 1.30)
*50–69*	2.57 (2.37, 2.82)	1.00 (0.92, 1.10)	5.21 (4.65, 5.80)	1.04 (0.92, 1.15)	0.10 (0.00, 0.20)
*≥70*	2.07 (1.93, 2.26)	3.29 (3.06, 3.58)	6.75 (5.76, 7.40)	4.21 (3.59, 4.62)	0.90 (0.70, 1.00)
Female	11.01 (10.13, 12.29)	0.60 (0.55, 0.67)	17.91 (15.83, 19.73)	0.74 (0.66, 0.82)	0.70 (0.70, 0.80)
*5–14*	0.07 (0.05, 0.08)	0.02 (0.01, 0.02)	0.04 (0.03, 0.05)	0.01 (0.01, 0.01)	−1.60 (−2.00, −1.10)
*15–49*	1.54 (1.25, 1.82)	0.16 (0.13, 0.19)	1.82 (1.52, 2.13)	0.15 (0.13, 0.18)	−0.20 (−0.50, 0.10)
*50–69*	4.10 (3.77, 4.64)	1.56 (1.43, 1.76)	6.00 (5.24, 6.69)	1.15 (1.01, 1.29)	−1.00 (−1.10, −0.90)
*≥70*	5.31 (4.81, 5.93)	5.58 (5.05, 6.23)	10.05 (8.53, 11.02)	4.85 (4.12, 5.32)	−0.50 (−0.60, −0.40)

*Expressed in years.

AAPC – average annual percent change, ASMR – age-standardised mortality rate, G20 – Group of Twenty, UI – uncertainty interval

**Table3:** The DALYs cases, age-standardised rates, and temporal trend of thyroid cancer from 1990 to 2019

Characteristics	1990		2019		1990–2019
	**DALYs**	**ASDR per 100 000**	**DALYs**	**ASDR per 100 000**	**AAPC**
	**No. ×10^3^ (95% UI)**	**No. (95% UI)**	**No. ×10^3^ (95% UI)**	**No. (95% UI)**	**No. (95% UI)**
China*					
Both	103.49 (87.96, 124.72)	8.74 (7.43, 10.54)	187.32 (156.24, 219.11)	13.17 (10.98, 15.40)	1.40 (1.10, 1.70)
*5–14*	4.27 (3.08, 4.91)	2.06 (1.49, 2.37)	1.91 (1.49, 2.22)	1.33 (1.04, 1.55)	−1.40 (−2.40, −0.30)
*15–49*	38.51 (30.66, 45.95)	5.76 (4.59, 6.87)	43.53 (36.39, 51.85)	6.04 (5.05, 7.19)	0.20 (−0.40, 0.90)
*50–69*	43.04 (36.40, 52.96)	27.94 (23.63, 34.38)	87.32 (71.95, 103.65)	23.67 (19.51, 28.10)	−0.60 (−0.90, −0.30)
*≥70*	17.67 (15.36, 24.11)	46.18 (40.15, 63.01)	54.56 (45.32, 62.92)	50.53 (41.97, 58.28)	0.30 (0.10, 0.60)
Male	38.44 (31.36, 46.79)	6.30 (5.14, 7.67)	107.11 (81.45, 133.26)	14.78 (11.24, 18.39)	2.90 (2.70, 3.20)
5–14	1.89 (1.53, 2.21)	1.76 (1.43, 2.06)	1.17 (0.83, 1.43)	1.51 (1.07, 1.84)	−0.60 (−1.00, −0.10)
15–49	13.21 (10.65, 16.01)	3.82 (3.08, 4.63)	25.10 (18.92, 30.98)	6.79 (5.12, 8.38)	2.00 (1.60, 2.40)
50–69	17.21 (13.63, 21.60)	21.57 (17.08, 27.07)	49.66 (37.33, 62.99)	26.92 (20.24, 34.14)	0.80 (0.50, 1.10)
≥70	6.13 (5.10, 8.16)	37.12 (30.90, 49.43)	31.17 (23.65, 37.84)	63.31 (48.03, 76.86)	1.90 (1.50, 2.30)
Female	65.05 (52.36, 80.81)	11.34 (9.13, 14.09)	80.21 (64.74, 99.49)	11.50 (9.28, 14.26)	0.00 (−0.30, 0.40)
*5–14*	2.38 (1.39, 2.93)	2.38 (1.39, 2.93)	0.74 (0.62, 0.85)	1.12 (0.94, 1.29)	−2.50 (−3.90, −1.10)
*15–49*	25.30 (18.55, 32.16)	7.83 (5.74, 9.96)	18.43 (14.26, 24.16)	5.25 (4.06, 6.88)	−1.50 (−2.50, −0.50)
*50–69*	25.83 (20.73, 33.39)	34.79 (27.92, 44.96)	37.66 (29.89, 47.02)	20.42 (16.21, 25.50)	−1.80 (−2.30, −1.40)
*≥70*	11.54 (9.57, 16.71)	53.05 (43.99, 76.81)	23.38 (18.86, 28.62)	39.82 (32.12, 48.73)	−1.00 (−1.20, −0.90)
**G20**					
Both	464.98 (432.00, 513.06)	12.56 (11.67, 13.86)	806.22 (733.39, 869.47)	16.58 (15.08, 17.88)	1.00 (0.80, 1.10)
*5–14*	10.55 (8.71, 11.90)	1.49 (1.23, 1.68)	7.35 (6.55, 8.27)	1.10 (0.98, 1.23)	−1.00 (−1.40, −0.60)
*15–49*	137.70 (119.84, 157.03)	7.11 (6.19, 8.10)	199.17 (176.79, 220.00)	8.03 (7.13, 8.87)	0.50 (0.30, 0.60)
*50–69*	207.51 (194.88, 225.35)	39.92 (37.49, 43.35)	362.15 (328.09, 391.33)	35.43 (32.10, 38.28)	−0.40 (−0.50, −0.30)
*≥70*	109.23 (101.89, 120.52)	69.06 (64.42, 76.20)	237.54 (210.04, 255.34)	64.67 (57.18, 69.51)	−0.30 (−0.30, −0.20)
Male	164.92 (152.22, 181.44)	8.82 (8.14, 9.70)	354.76 (315.15, 390.69)	14.49 (12.87, 15.96)	1.70 (1.60, 1.80)
*5–14*	4.96 (4.28, 5.77)	1.36 (1.17, 1.58)	3.85 (3.36, 4.35)	1.10 (0.96, 1.24)	−0.70 (−1.10, −0.30)
*15–49*	47.94 (43.38, 54.03)	4.85 (4.39, 5.46)	85.48 (75.42, 95.15)	6.75 (5.96, 7.52)	1.20 (0.90, 1.40)
*50–69*	80.35 (74.09, 88.35)	31.33 (28.89, 34.45)	167.80 (148.22,186.20)	33.40 (29.50, 37.06)	0.20 (0.10, 0.30)
*≥70*	31.67 (29.53, 34.51)	50.24 (46.84, 54.74)	97.62 (85.05, 106.68)	60.90 (53.06, 66.55)	0.70 (0.50, 0.80)
Female	300.07 (269.57, 338.36)	16.38 (14.71, 18.47)	451.46 (398.87, 500.97)	18.69 (16.51, 20.74)	0.50 (0.30, 0.60)
*5–14*	5.59 (4.12, 6.67)	1.63 (1.20, 1.94)	3.50 (3.05, 4.03)	1.09 (0.95, 1.26)	−1.40 (−1.80, −0.90)
*15–49*	89.75 (73.14, 104.59)	9.46 (7.71, 11.03)	113.69 (95.58, 130.71)	9.36 (7.87, 10.77)	0.00 (−0.30, 0.30)
*50–69*	127.16 (116.00, 142.89)	48.29 (44.05, 54.26)	194.35 (169.76, 215.90)	37.39 (32.66, 41.53)	−0.90 (−1.00, −0.70)
*≥70*	77.56 (71.44, 86.99)	81.54 (75.11, 91.46)	139.92 (121.50, 152.60)	67.58 (58.68, 73.70)	−0.60 (−0.80, 0.50)

*Expressed in years.

AAPC – average annual percent change, ASDR – age standardised DALYs rate, DALYs – disability-adjusted life years, G20 – Group of Twenty, UI – uncertainty interval

**Table 4:** The MIR and temporal trend of thyroid cancer from 1990 to 2019

Characteristics	1990	2019	1990–2019
	**MIR**	**MIR**	**AAPC**
			**No. (95% UI)**
China*			
Both	0.33	0.19	−2.00 (−2.20, −1.80)
*5–14*	0.18	0.05	−4.00 (−4.30, −3.70)
*15–49*	0.14	0.05	−3.60 (−3.90, −3.30)
*50–69*	0.40	0.15	−3.20 (−3.30, −3.10)
*≥70*	0.93	0.64	−1.30 (−1.40, −1.20)
Male	0.49	0.26	−2.20 (−2.40, −1.90)
*5–14*	0.26	0.07	−4.20 (−4.30, −4.10)
*15–49*	0.25	0.08	−3.90 (−4.10, −3.70)
*50–69*	0.53	0.21	−3.20 (−3.40, −3.00)
*≥70*	1.03	0.77	−1.00 (−1.00, −0.90)
Female	0.28	0.13	−2.50 (−2.70, −2.30)
*5–14*	0.14	0.04	−4.60 (−4.70, −4.50)
*15–49*	0.11	0.03	−4.50 (−4.60, −4.30)
*50–69*	0.34	0.11	−3.70 (−3.80, −3.60)
*≥70*	0.89	0.51	−1.90 (−2.00, −1.80)
**G20**			
Both	0.24	0.19	−0.90 (−1.00, −0.90)
*5–14*	0.13	0.07	−2.30 (−2.50, −2.10)
*15–49*	0.09	0.06	−1.70 (−1.90, −1.60)
*50–69*	0.23	0.15	−1.40 (−1.50, −1.30)
*≥70*	0.61	0.51	−0.60 (−0.70, −0.50)
**Male**	0.29	0.23	−0.80 (−0.90, −0.70)
*5–14*	0.18	0.09	−2.40 (−2.60, −2.20)
*15–49*	0.13	0.08	−1.60 (−1.80, −1.40)
*50–69*	0.29	0.19	−1.40 (−1.50, −1.30)
*≥70*	0.65	0.57	−0.50 (−0.50, −0.40)
**Female**	0.22	0.16	−1.10 (−1.20, −1.00)
*5–14*	0.10	0.05	−2.40 (−2.60, −2.20)
*15–49*	0.08	0.04	−2.00 (−2.10, −1.90)
*50–69*	0.20	0.13	−1.60 (−1.70, −1.40)
*≥70*	0.59	0.48	−0.80 (−0.80, −0.70)

*Expressed in years.

AAPC – average annual percent change, G20 – Group of Twenty, MIR – mortality-incidence ratio, UI – uncertainty interval

### Gender and age disparities of thyroid cancer between China and the G20 countries

The incidence case and ASIR were predominantly higher in women than in men in both China and the G20 countries. However, the AAPC of ASIR was more pronounced in men (**Table 1**, **Figure 1**). In contrast, the mortality case and ASMR were higher in men in China, while the situation was reversed in the G20 countries (**Table 2**). Additionally, the trend in DALY mirrored the mortality trend (**Table 3**).

The group aged 15–49 and 50–69 constituted the vast majority of incidence cases in both China and the G20 countries (**Figure 1**, panels A, C). Within these age groups, the proportion of incidence cases among the group aged 50–69 displayed a fluctuating upward trend from 1990 to 2019, whereas the other groups exhibited a declining trend during the same period (**Figure 1**, panels A, C). Although China shared a similar trend of change with the G20 countries, the extent of change was more pronounced in China (**Table 1**). Although the group aged over 70 had the highest proportion of mortality cases, which consistently increased from 1990 to 2019, their proportion among incidence cases remained relatively low and stable (**Figure 1**, panels B, D). Interestingly, the 50–69 age group, while showcasing an increase in the proportion of incidence cases, revealed a decline in the proportion of mortality cases, particularly among men (**Table 2**, **Figure 1**, panels B, D). Similarly, the proportion of DALY in the group of aged 50–69 decreased in China and the G20 countries over the period (**Table 3**).

### Trend in ASIR, ASMR and MIR of thyroid cancer

**Figure 2:**
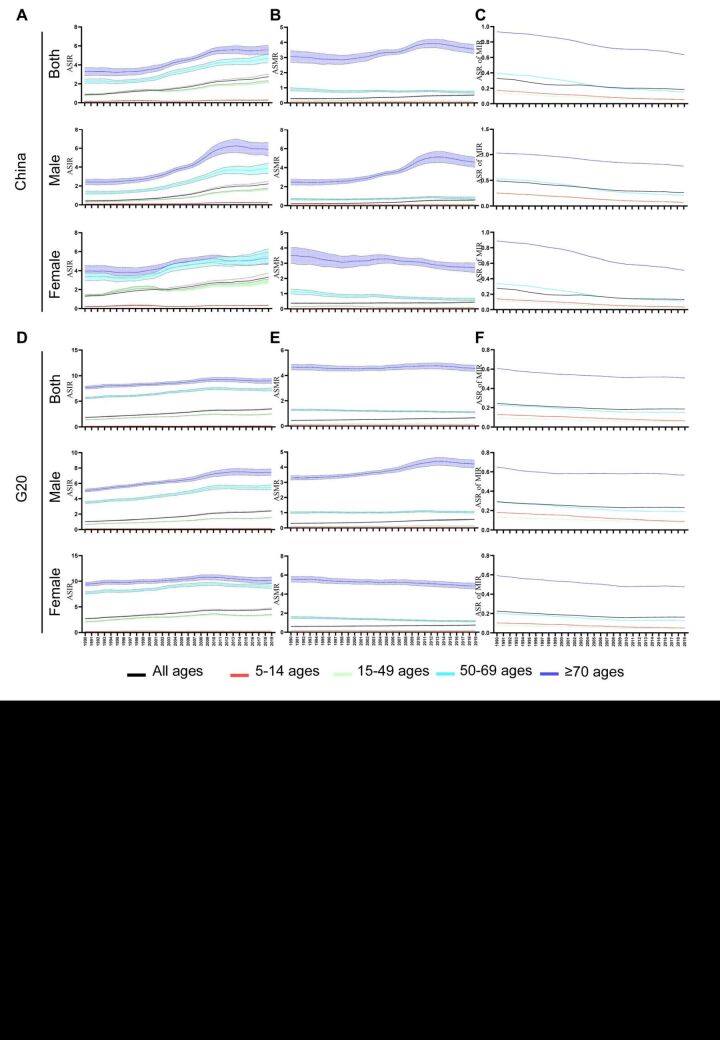
The trend of ASIR, ASMR and ASR of MIR of thyroid cancer. **Panels A–C.** The trend of ASIR (**A**), ASMR (**B**) and ASR of MIR (**C**) in different age group, genders in China from 1990 to 2019. **Panels D–F.** The trend of ASIR (**D**), ASMR (**E**) and ASR of MIR (**F**) in different age group, genders in the G20 countries. ASIR **–** age-standardised incidence rate, ASMR **–** age-standardised mortality rate, G20 – Group of Twenty, MIR **–** mortality-incidence ratio

The ASIR increased across all age groups and genders in China and the G20 countries, with a particularly pronounced rise observed among Chinese men (**Table 1**, **Figure 2**, panels A, D). The group in men aged 15–49 displayed the highest AAPC of ASIR in both China and the G20 countries. Furthermore, the growth rate of ASIR was more accentuated in men compared to women and was also more significant in China than in the G20 countries. In contrast to the trend of ASIR, the ASMR for women decreased across all age groups in both China and the G20 countries, especially in the group aged 5–14 and 50–69 (**Figure 2**, panels B, E). On the other hand, the ASMR significantly increased in the group aged over 70 in China and the G20 countries (**Table 2**). Additionally, China exhibited a higher increase in ASMR compared to the G20 countries, especially among men (**Figure 2**, panels B, E). Regarding the ASDR trend, it paralleled the ASMR trend. While the ASDR significantly decreased in the group aged 5–14, there was a notable increase in the group aged 50–59 and among those over 70 in China and the G20 countries from 1990 to 2019 (**Table 3**). Fortunately, both China and the G20 countries exhibited a declining trend in the MIR across all age groups and genders. Although the MIR declined, the decline in the group aged over 70 was significantly lower than that in other age groups. Moreover, the decline was less pronounced among men compared to women (**Table 4**, **Figure 2**, panels C, F).

### Joinpoint regression analysis of thyroid cancer ASR trend

**Figure 3:**
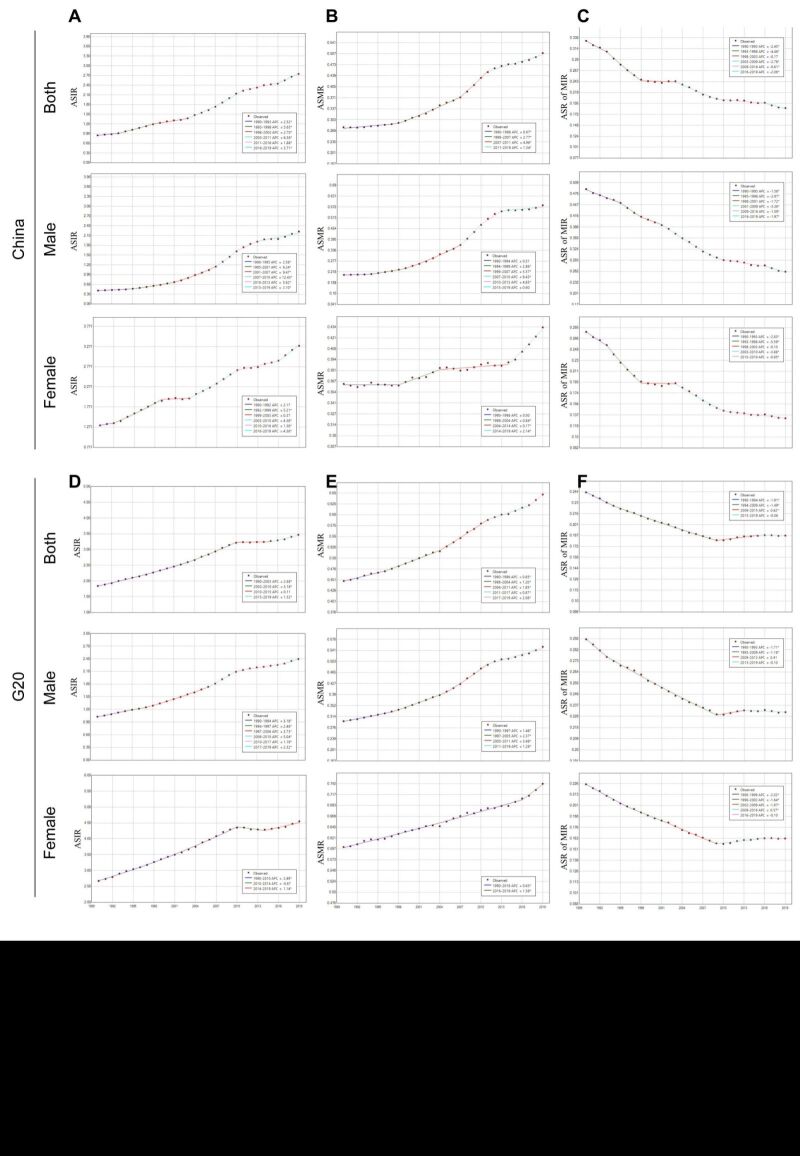
The joinpoint regression analysis of ASIR, ASMR and ASR of MIR in thyroid cancer. **Panels A–C.** Joinpoint regression analysis of ASIR (**A**), ASMR (**B**) and ASR of MIR (**C**) in thyroid cancer in China for genders from 1990 to 2019. **Panels D–F.** Joinpoint regression analysis of ASIR (**D**), ASMR (**E**) and ASR of MIR (**F**) in thyroid cancer in the G20 countries for genders from 1990 to 2019. ASIR – age-standardised incidence rate, ASMR – age-standardised mortality rate, G20 – Group of Twenty, MIR – mortality-incidence ratio

In China, notable shifts in ASIR were observed in 1993, 1998, 2003, 2011, and 2016, whereas in the G20 countries, significant changes were noted in 2003, 2010, and 2015 (**Figure 3**, panels A, D). The most substantial increase in ASIR in China was observed during 2003–2011, followed by 1993–1998. In contrast, in the G20 countries, the most significant increase occurred during 2003–2010. Additionally, the overall ASMR trends in both China and the G20 countries exhibited an upward trajectory, with significant inflection points in 1998, 2007, and 2011 in China, and 1996, 2004, 2011, and 2017 in the G20 countries (**Figure 3**, panels B, E). The period with significant change in China was 2007–2010 in men and 2014–2019 in women, and in the G20 countries was 2005–2011 in men (**Figure 3**, panels B, E). In contrast to the trend of ASIR and ASMR, the MIR demonstrated a downward trend. Significant changes were observed in 1993, 1998, 2003, and 2016 in China, and in 1994, 2009, 2015 in the G20 countries (**Figure 3**, panels C, F). Interestingly, while the MIR for Chinese women had been consistently decreasing, the rate of this decline decelerated post 2010. On the other hand, the MIR for women in the G20 countries had remained stable since 2009 (**Figure 3**, panels C, F).

Our analysis highlighted that the 15–49 age group constituted the majority of the thyroid cancer population, with the ASR showcasing the most rapid growth. To further understand this trend, we employed joinpoint regression analysis. The ASIR increased throughout the observed period in China, especially between 2006 and 2010 (Figure S1 in the **Online Supplementary Document**). Remarkably, among Chinese women in this age group, the ASIR showed a significant increase from 1993 to 2000, followed by a swift decline from 2000 to 2003 (Figure S1 in the **Online Supplementary Document**). The ASMR for this age group in China showed varying declines, while in the G20 countries, it displayed oscillating increases (Figure S1 in the **Online Supplementary Document**).

### Prediction of ASIR and ASMR of thyroid cancer

**Figure 4:**
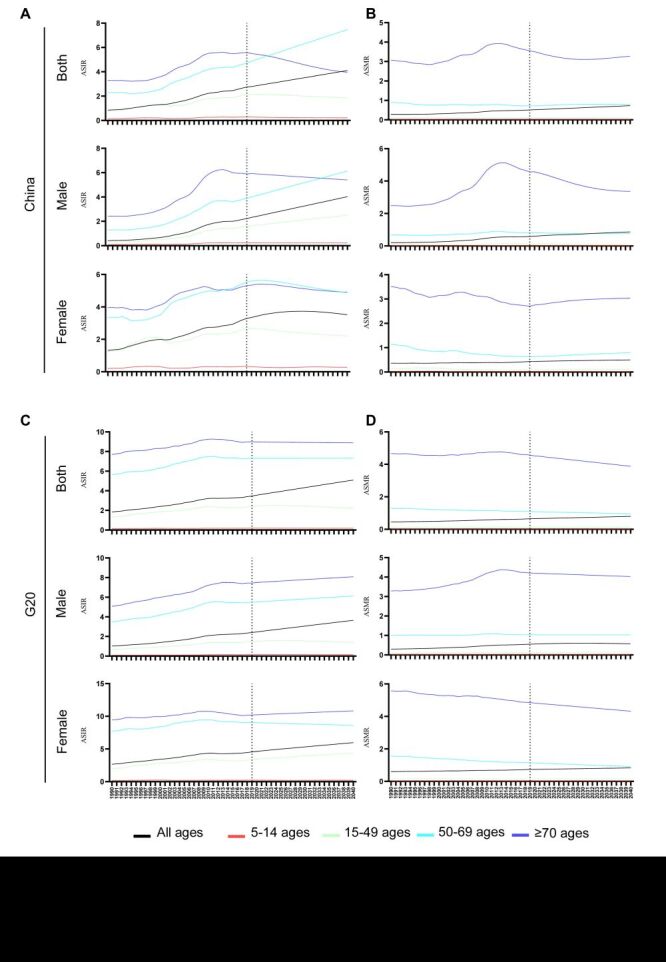
Prediction of ASIR and ASMR of thyroid cancer. **Panels A–B.** Prediction of ASIR (**A**) and ASMR (**B**) of thyroid cancer in different age groups and genders in China until 2040. **Panels C–D.** Prediction of ASIR (**C**) and ASMR (**D**) of thyroid cancer in different age groups and genders in the G20 countries. ASIR – age-standardised incidence rate, ASMR – age-standardised mortality rate, G20 – Group of Twenty

The ASIR is predicted to increase by 49.1% from 2019 to 2040, with the most significant increase expected in the group aged 50–69 (**Table 5**, **Figure 4**, panels A, C). In contrast, the group aged over 70 is expected to see a decline in ASIR in China, while remaining stable in the G20 countries (**Figure 4**, panels A, C). Over the upcoming two decades, the ASIR for women in China is predicted to stabilise, whereas an upward trend is foreseen for the G20 countries (**Figure 4**, panels A, C). Moreover, a decline in ASIR for men is projected only for the group aged over 70 in China and for the age group aged 5–14 and 14–49 in the G20 countries (**Figure 4**, panels A, C).

**Table 5:** The prediction of mortality and incidence ASR of thyroid cancer in 2040

Characteristics	2040	
	**ASIR per 100 000**	**ASMR per 100 000**
	**No.**	**No.**
China*		
Both	4.10	0.73
*5–14*	0.22	0.02
*15–49*	1.86	0.11
*50–69*	7.48	0.80
*≥70*	3.96	3.27
Male	4.03	0.86
*5–14*	0.23	0.02
*15–49*	2.50	0.10
*50–69*	6.13	0.76
*≥70*	5.40	3.36
Female	3.52	0.50
*5–14*	0.27	0.02
*15–49*	2.21	0.12
*50–69*	4.88	0.79
*≥70*	4.90	3.03
**G20**	5.10	0.79
Both		
*5–14*	0.18	0.01
*15–49*	2.21	0.14
*50–69*	7.31	0.96
*≥70*	8.88	3.88
Male	3.65	0.57
*5–14*	0.13	0.01
*15–49*	1.38	0.15
*50–69*	6.13	1.03
*≥70*	8.08	4.03
Female	5.97	0.83
*5–14*	0.22	0.02
*15–49*	4.41	0.16
*50–69*	8.61	0.91
*≥70*	10.83	4.32

*Expressed in years.

ASIR – age standardised incidence rate, ASMR – age standardised mortality rate, ASR – age standardised rate, G20 – Group of Twenty

The ASMR is also predicted to increase over the next 20 years. Notably, the group aged over 70, which records the highest proportion of thyroid cancer fatalities, is predicted to experience a reduction in their ASMR by 2040 (**Table 5**, **Figure 4**, panels B, D). Additionally, while the ASMR for men in the 15–49 age group in China is anticipated to decrease, an increase is expected for women in China and for both genders in the G20 countries (**Figure 4**, panels B, D).

### The trend of thyroid cancer deaths attributable to risk factor in the G20 countries

**Figure 5:**
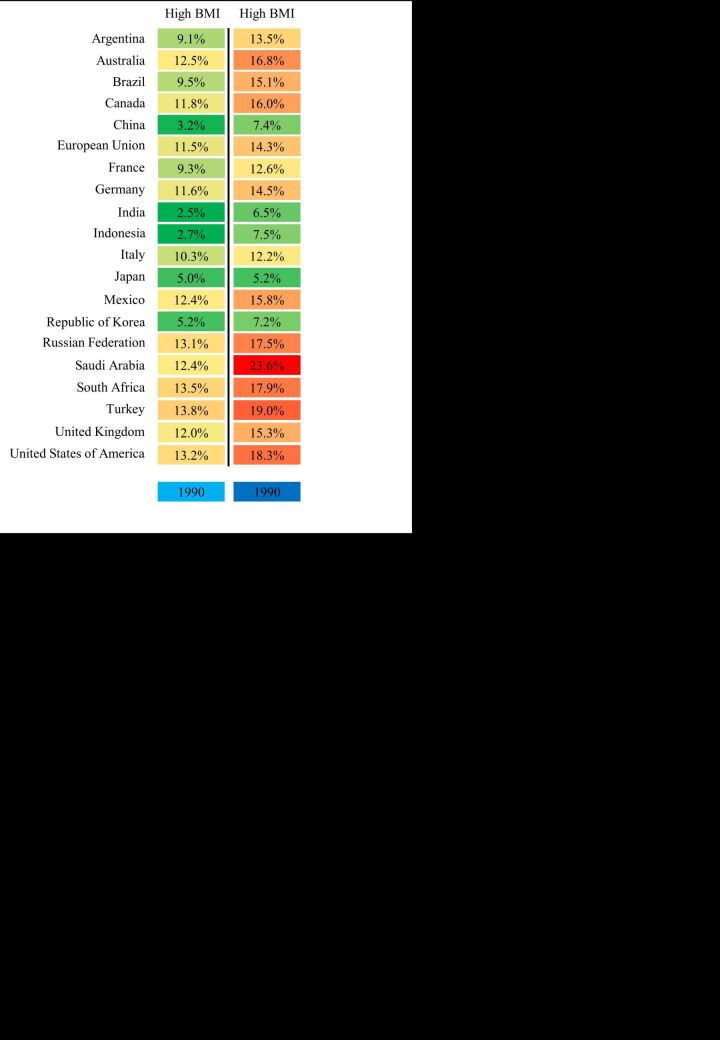
The attributable risk factors of thyroid cancer in the G20 countries in 1990 and 2019. BMI – body mass index, G20 – Group of Twenty

The percentage of thyroid cancer deaths attributable to high body mass index (BMI) exhibited an escalating trend from 1990 to 2019, with variations across the G20 countries. Saudi Arabia recorded the most substantial rise from 12.4% in 1990 to 23.6% in 2019 (**Figure 5**). Similarly, China also witnessed an increase, with this percentage rising from 3.2 to 7.4% (**Figure 5**). Among the G20 countries, Japan had the least pronounced increase over this period (**Figure 5**).

### Association between AAPC of ASIR, ASMR and SDI

**Figure 6:**
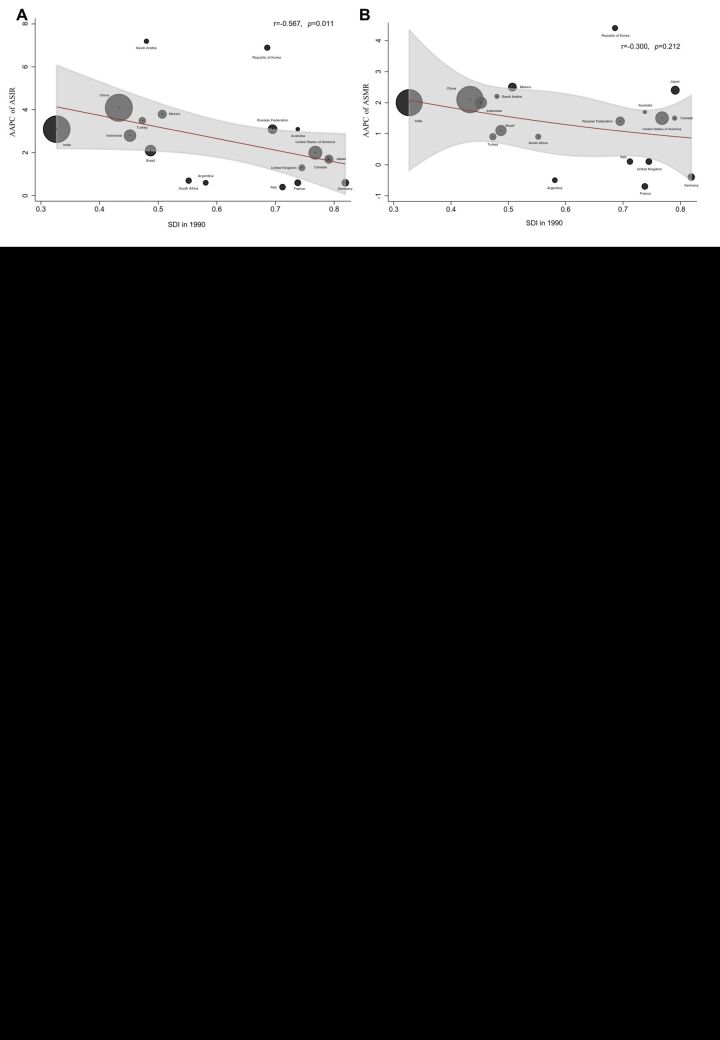
The correlation between SDI and AAPC of ASIR and ASMR in the G20 countries. **Panel A.** The correlation between SDI and AAPC of ASIR. **Panel B.** The correlation between SDI and AAPC of ASMR. AAPC – average annual percent change, ASIR – age-standardised incidence rate, ASMR – age-standardised mortality rate, G20 – Group of Twenty, SDI – sociodemographic index

The analysis revealed a negative relationship between SDI and AAPC of ASIR (r = 0.567, *P* = 0.011). Notably, Sandi Arabic, Republic of Korea exhibited a higher AAPC of ASIR compared to countries with analogous SDI (**Figure 6**, panel A). In contrast, South Africa, Argentina and Italy obtained a lower AAPC than the average level (**Figure 6**, panel A). While the AAPC of the ASMR tended to decrease as SDI increased, no statistically significant correlation was found between them (**Figure 6**, panel B). Among the G20 countries, Japan obtained a higher AAPC than the average, while France, Argentina, Italy, and UK showcased a lower AAPC than the average (**Figure 6**, panel B).

## DISCUSSION

Thyroid cancer is emerging as a significant global health challenge, characterised by its increasing incidence and mortality cases. The GBD study allows us to study the disease burden, predict and compare the disease trend [27]. In our study, we initially examined and compared the trend of thyroid cancer in incidence, mortality and MIR in China and the G20 countries. Additionally, we made predictions for the next two decades, considering various age groups, genders, and countries. These findings hold the potential to inform resource allocation strategies and the development of effective intervention measures.

Our study revealed a continuous increase in thyroid cancer incidence over the past 30 years, with projections indicating further increases in the next 20 years. The advancements in imaging examinations, ultrasonic examinations, and biopsies have significantly contributed to overdiagnosis [3,28,29]. Additionally, the promotion of cancer screening programmes has further exacerbated the issue [30]. However, recent thyroid cancer guidelines have emphasised the importance of active surveillance, particularly for patients with T1N0M0 thyroid cancer [31]. Unfortunately, due to insufficient awareness of active surveillance, some patients who could benefit from this approach have been subjected to overdiagnosis and overtreatment. With the expansion of active surveillance, stricter criteria for biopsies, and updates to guidelines, the continuously increasing incidence of thyroid cancer may be curbed in the future. While overdiagnosis and overtreatment likely contribute to the increasing trend of thyroid cancer, it is important to note that these factors may not fully account for the upward trajectory [32]. A noteworthy shift occurred in 2015 when the American Thyroid Association (ATA) revised its management guidelines, adopting a more conservative approach to the diagnosis and treatment of thyroid cancer [33]. However, the incidence has not decreased but has continued to rise after 2015. A study conducted by Lim also reported an increase in thyroid cancer incidence and mortality among advanced-stage PTC patients in the US since 1974, indicating a true increase in the incidence [34]. Therefore, we should pay more attention to other risk factors, such as environmental factors, genetic factors, obesity [35,36]. Our study identified a deceleration in the rising trend of thyroid cancer incidence from 2011 to 2016 in China and from 2010 to 2015 in the G20 countries. This deceleration could be attributed to increased awareness of education and disease prevention during this period, coupled with improved iodine supplementation practices [37]. Previous study indicated that variations in iodine intake might not directly impact the risk of thyroid cancer but might result in thyroid cancer transforming into less malignant subtypes [38]. In China, iodized salt was promoted nationwide in 1996. These might provide a potential explanation for the decreasing trend of incidence during this specific timeframe.

In term of genders, the ASIR in China and the G20 countries, ASMR in the G20 countries, ASDR in the G20 countries were all higher in women than that in men. However, the AAPC of ASIR, ASMR, ASDR and ASR of MIR were all higher in men than in women. Previous studies indicated that gender, age, race and ethnicity were the most risk factors for thyroid cancer [5]. Additionally, women had a 3-fold higher incidence than men in thyroid cancer [34]. One reason for this difference was the variation in the screening opportunities available to different genders. Women were more likely to seek and make use of health care than men, whereas men were more frequently diagnosed at older ages and at more advanced PTC [39,40]. Moreover, the risk factors were different between genders. A study indicated that in utero exposure may increase the risk of thyroid cancer in offspring [41]. Another study suggested a strong association of parity (≥3 pregnancies) with the risk of thyroid cancer, and recent pregnancy was also related with transient increased risk of thyroid cancer [42]. A recent study discovered that oestrogen promoted growth of thyroid cancer through classical genomic pathways and non-genomic pathways, mediated by membrane-bound oestrogen receptors. Several signal pathways involved in the process, such as AKT/mTOR pathway, ERK pathway, VEGF pathway, and etc [43]. Moreover, studies also demonstrated that thyroid autoimmune were risk factors for women, while smoking and alcohol consumption were protected factors for men [44–48]. Obesity represented another significant risk factor for thyroid cancer, and it had a substantially greater impact on men than on women. To reduce the AAPC of ASR, additional medical screening opportunities for men are advisable.

In term of age, the ASIR increased across all age groups, with the most significant rise occurring in the group aged 15–49. Several factors may contribute to this increase, including the widespread use of ultrasound and fine-needle aspiration biopsy and the overall increase in medical exposure [49,50]. Similar to our findings, studies also found that the thyroid cancer incidence in women increased rapidly after the age of 15, and reached a peak at the age of 40–45 [37,51]. A meta-analysis also found that the menopausal age in women was an independent risk factor for thyroid cancer [52]. Moreover, the significant increase in ASIR among group aged 15–49 was also closely associated with their level of education and their awareness of health and disease [53]. Moreover, a study demonstrated that the highest percentage of CT scans, identified as a risk factor for thyroid cancer, occurred in individuals aged 36–50 [54]. The RET/PTC rearrangement in thyroid tumours was found to be more prevalent in children and young adults [55]. The ASMR decreased in women across all age groups, but significantly increased in the group aged over 70. Furthermore, the decline in ASR of MIR among the group aged over 70 was more substantial compared to other age groups. Similar findings had been replicated in various other studies, and this trend might be attributed to the following factors [9,10]. First, improvements in medical care have extended the survival time of cancer patients. Second, the aging of the population has led to an increase in the elderly population, which has subsequently affected the mortality of thyroid cancer [56]. Third, previous study indicated that thyroid cancer patients in men were diagnosed later and at a more advanced stage, resulting in a poorer prognosis [40]. Four, in the past, due to limitations in health care conditions, some elderly people died at home and were not included in the statistics. However, there has been a notable increase in the number of elderly people dying in hospitals, further affecting the mortality [10]. Among thyroid cancer patients aged over 70, the risk increases significantly, and this increase was not liner in nature [10]. Fortunately, while the ASIR and ASMR are predicted to increase in the future, the group aged 70 is exhibiting a decrease trend both in ASIR and ASMR over the next 20 years. This positive trend is closely linked to expanded health care coverage for the population, enhancements in thyroid cancer management, and improvements in population demographics.

Obesity represents a significant risk factor for thyroid cancer, particularly among women [57]. A study revealed that individuals who were overweight faced a 25% higher risk of developing thyroid cancer compared to those with a normal body weight, while obese individuals had a 55% increased risk [58]. In our study, the percentage of thyroid cancer deaths attributable to high BMI escalated in the G20 countries from 1990 to 2019. This phenomenon can be attributed to various factors. First, economic growth and improvements in quality of life have contributed to the increasing prevalence of obesity. A study found that in low-income countries, obesity mainly affected middle-aged women from affluent urban areas, whereas in high-income countries, obesity affected all population groups, with a more significant impact on vulnerable populations [59]. Second, changes in dietary habits, the growing consumption of processed foods, and the emergence of high-calorie junk food have also played a role in the obesity epidemic [7,60]. Interestingly, our study uncovered differences in the attributable mortality rates between Asian countries (e.g. Japan, China) and Western countries, with the former experiencing lower rates and the latter witnessing a faster increase. Dietary habits are a significant contributing factor to this disparity [7]. Fortunately, as awareness of the risks associated with obesity has grown, there has been a notable deceleration in its increasing trend, particularly in developed countries [61]. By promoting better dietary choices and more effective weight management, it is possible to reverse the increasing trend of thyroid cancer in the future.

In the relationship analysis, our study identified that Sandi Arabic exhibited a higher AAPC of ASIR compared to countries with analogous SDI scores. Over the past three decades, Saudi Arabia has undergone significant demographic, economic, and cultural changes, all of which have had an impact on health management strategies, including those related to thyroid cancer. Alongside well-established risk factors such as lifestyle changes, radiation exposure and obesity, long-term exposure to particulate-laden dust storms and environmental air pollution have contributed to an increased risk of thyroid cancer [62]. In addition, Korea exhibited a higher AAPC of ASIR and ASMR than other countries with similar SDI scores. It was closely related to Korea’s introduction of free cancer screening in 1999. Cancer screening not only leads to a significant increase in the incidence of thyroid cancer, but also results in more neck examinations, exposing the population to additional medical-related radiation [63]. Moreover, population aging in Korea also influences the trend of thyroid cancer [64].

Our study is subject to several limitations that should be considered when interpreting the findings. First, the accuracy of our findings relies on the quality of the original data from the GBD study. However, the GBD study lacks population data from remote areas, and differences in diagnostic criteria can introduce bias into the analysis. This geographic and diagnostic inconsistency could distort our understanding of the disease's actual impact, especially in underserved regions. This might result in the misallocation of health care resources or biased assessments of risk factors. Second, thyroid cancer has numerous risk factors, including obesity, genetics, radiation exposure, endocrine-disrupting chemicals, and more. However, the GBD study only includes obesity as one of these risk factors for thyroid cancer. This omission might lead to an incomplete analysis of the disease's aetiology, compromising the development of comprehensive prevention and intervention strategies that encompass all significant risk factors. Third, the thyroid cancer data in GBD study does not include information on pathological types or clinical staging. Such data could offer insights into disease severity, prognosis, and the effectiveness of different treatment modalities. Without this information, research and health care strategies might not be optimally tailored to the specific characteristics of thyroid cancer, affecting the study's applicability in clinical settings.

## CONCLUSIONS

The escalating incidence of thyroid cancer poses a substantial global burden, particularly due to the challenges of overdiagnosis and overtreatment, which can exert long-term effects on patients. It is important to promote active surveillance, establish stricter biopsy criteria, and timely update of guidelines to reduce overdiagnosis and overtreatment. Moreover, it's crucial to deliberate on and identify the target population for thyroid cancer screening, as opposed to advocating for universal screening. Although thyroid cancer is a global public health concern, its trends vary among the G20 countries. Recognising the risk factors which cause these variations is essential for the development of tailored strategies. Measures targeting obesity, iodine intake, and thyroiditis might reduce the incidence and the unnecessary use of medical resources. Additionally, focusing on high-risk populations through targeted surveillance can help alleviate the growing disease burden effectively.

## Additional material


Online Supplementary Document
