# Supplementary material for: Thyroid cancer trends in China and its comparative analysis with G20 countries: Projections for 2020–2040
Source: J Glob Health. 2024 Jun 14;14:04131. doi: 10.7189/jogh.14.04131 (PMC11177899; doi:10.7189/jogh.14.04131)
Supplement: Online Supplementary Document [file jogh-14-04131-s001.pdf]

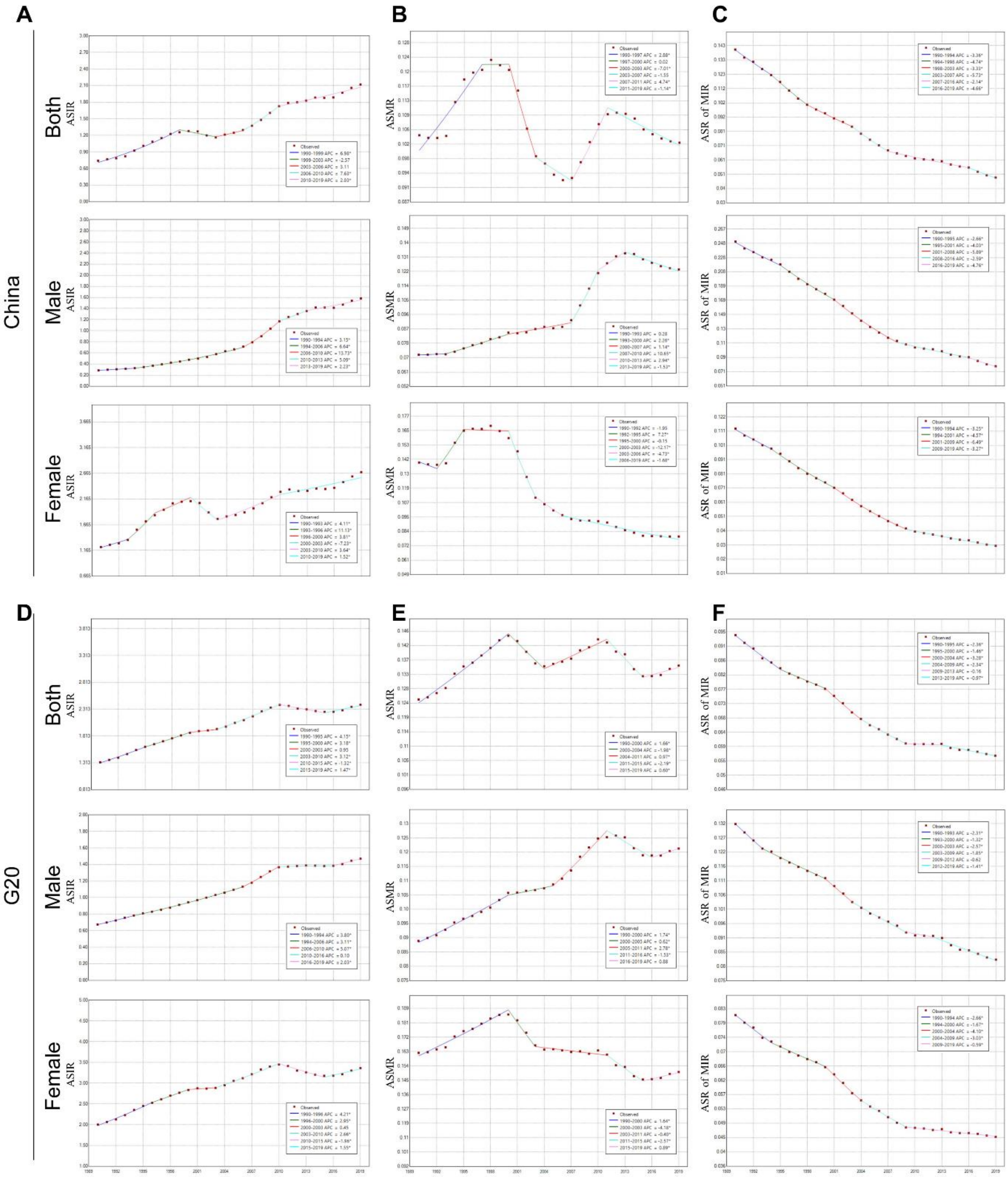

Figure S1. The joinpoint regression analysis of ASIR, ASMR and ASR of MIR in the group aged 15–49. **Panels A–C.** Joinpoint regression analysis of ASIR

(A), ASMR (B) and ASR of MIR (C) in thyroid cancer in China for genders from 1990 to 2019 in the group aged 15–49. **Panels D–F.** Joinpoint regression analysis of ASIR (D), ASMR (E) and ASR of MIR (F) in thyroid cancer in the G20 countries for genders from 1990 to 2019 in the group aged 15-49. ASIR – age-standardised incidence rate, ASMR – age-standardised mortality rate, MIR – mortality-incidence ratio
